# NAD^+^ to assess health in aging humans

**DOI:** 10.18632/aging.204220

**Published:** 2022-08-06

**Authors:** Georges E. Janssens, Riekelt H. Houtkooper, Joris Hoeks

**Affiliations:** 1 Laboratory Genetic Metabolic Diseases, Amsterdam University Medical Centers, University of Amsterdam, Amsterdam, The Netherlands; 2 Amsterdam Gastroenterology, Endocrinology and Metabolism Institute, Amsterdam University Medical Centers, University of Amsterdam, Amsterdam, The Netherlands; 3 Amsterdam Cardiovascular Sciences Institute, Amsterdam University Medical Centers, University of Amsterdam, Amsterdam, The Netherlands; 4 Department of Nutrition and Movement Sciences, NUTRIM School of Nutrition and Translational Research in Metabolism, Maastricht University, Maastricht, The Netherlands

**Keywords:** NAD ^+^, healthspan, exercise, aging, frailty

The molecular changes that occur in aging cells prime the organism for age-related disease. One of the changes that occur with age, extensively documented in mice, is the decline of nicotinamide adenine dinucleotide (NAD^+^) [1]. Discovered over 100 years ago as the electron carrier for redox enzymes, and later revealed as a signaling hub, NAD^+^ has received a surge of attention in the last decades in the aging research field [1]. Over 500 enzymatic reactions use NAD^+^ to sustain life and cellular homeostasis, and supplementing mice with NAD^+^ precursors improves a plethora of pathways that get derailed during aging, including gluconeogenesis in the liver, insulin secretion in the pancreas, immune functioning in lymphoid tissues, cardioprotection in the heart, sensory and motor function in the brain, and many more, including lifespan extension [1,2].

While the evidence has looked compelling in preclinical worm and rodent models, little evidence existed to corroborate the role of NAD^+^ in healthy aging in humans [3]. Recently however, we performed an unbiased metabolomics study on skeletal muscle biopsies from young and older adults and found muscle NAD^+^ levels to be lower in aged humans compared to young [4,5]. Importantly, the older adults in our study displayed similar physical activity levels to the younger control group, ensuring that lower NAD^+^ levels were not due to the lower activity levels that generally co-occur with aging. Remarkably, the difference in muscle NAD^+^ was exacerbated in physically impaired older adults, while athletic older adults had NAD^+^ levels similar to those found in the young [4].

With this, our study added the much-needed confirmation in humans that NAD^+^ inherently declines with age in a major tissue such as skeletal muscle and that this is linked to (muscle) health, since physically impaired older adults had even lower levels of NAD^+^ than older adults with normal physical fitness [4]. Likewise, our study demonstrated that a lifestyle including sufficient aerobic exercise training was associated with NAD^+^ levels similar to those found in the young [4]. This is in line with another study demonstrating that twelve weeks of aerobic and resistance exercise in older adults could increase levels of nicotinamide phosphoribosyltransferase (NAMPT), the rate limiting enzyme of the NAD^+^ salvage pathway [6]. Physical activity and NAD^+^ levels were so strikingly linked in our own study that a direct correlation was observed between an individual’s average daily step count and their muscle NAD^+^ content [4].

NAD^+^, while the most striking, was not the only metabolite linked to the health status of the older adults. In fact, nearly all muscle metabolites we measured that were impacted by aging, followed a similar trend: the difference was less pronounced in the trained older adults and more severe in the physically impaired older adults. Although our study cannot reveal cause and consequence due to its cross-sectional nature, it supports the notion that exercise is one of the most powerful healthy aging interventions available to date [7]. Human intervention studies evaluating metabolite changes in skeletal muscle before and after exercise training in the older population would provide the direct evidence to support this idea. Furthermore, pairing exercise with NAD^+^ precursor supplementation may hold additive beneficial effects that deserve merit to be further explored. Such interventions may then be most beneficial for physically impaired older adults characterized by the most severe NAD^+^ depletion. However, this target group does not easily volunteer for scientific research, which may mask the full potential of these interventions.

With NAD^+^ involved in so many and such diverse cellular functions, ranging from DNA repair to circadian rhythms, redox reactions to cell division, inflammation to epigenetics [1,2,8], it could be argued that NAD^+^ plays a key role in the regulation of almost all biological processes. With NAD^+^ so convincingly linked to health in preclinical models [1], now demonstrated to reflect health in older humans [4], we ask ourselves the following: For any given intervention to significantly improve healthspan, should it not also serve to maintain NAD^+^ levels and homeostasis? We suggest that for a range of healthy aging interventions in humans—certainly including exercise—NAD^+^ abundance may serve as the best molecular marker to understand efficacy.
